# Co-Creating Organisational Health Literacy: Formative Evaluation and Feasibility Testing of OHL-Act

**DOI:** 10.3390/ijerph23030391

**Published:** 2026-03-18

**Authors:** Camilla Klinge Renneberg, Anne Sofie Dydensborg Rasmussen, Maiken Meldgaard, Helle Terkildsen Maindal, Anna Aaby

**Affiliations:** 1 Department of Public Health, Aarhus University, 8000 Aarhus, Denmarkaaby@ph.au.dk (A.A.); 2 Institute for Health Transformation, Deakin University, Burwood 3125, Australia

**Keywords:** health literacy responsiveness, organisational health literacy, health disparities, equity, healthcare settings, implementation research, formative evaluation, feasibility study, assessment, co-creation

## Abstract

**Highlights:**

**Public health relevance—How does this work relate to a public health issue?**

- Organisational health literacy is critical for reducing health disparities formed and sustained in health systems.
- Although organisational health literacy is widely promoted as a public health strategy to address health literacy-related inequities, many existing tools lack evaluation; this study evaluates and refines a practical organisational health literacy approach through applications in practice.

**Public health significance—Why is this work of significance to public health?**

- This study provides insights into how co-creational organisational health literacy approaches can be evaluated and refined in healthcare settings.
- The findings highlight the feasibility of a structured, bottom-up approach to support organisational reflection and prioritisation of organisational health literacy initiatives.

**Public health implications—What are the key implications or messages for practitioners, policymakers and/or researchers in public health?**

- This study demonstrates the feasibility of using a structured, co-creational workshop approach to support organisational reflection on health literacy in healthcare settings.
- The findings emphasise the importance of organisational context, including leadership support and participant composition, when implementing an organisational health literacy approach.

**Abstract:**

Background: Organisational health literacy (OHL) is increasingly recognised as a system-level strategy to address health literacy-related inequities in healthcare, yet evaluation of practical OHL tools and frameworks remain limited. This study aimed to examine the implementation experiences of the Danish OS! to inform refinements, and to examine the feasibility of the refined version, renamed OHL-Act, in practice. Methods: A two-phase study guided by the RE-AIM framework was conducted. Phase 1 comprised a formative evaluation of OS! based on interviews from previous applications, informing refinement. Phase 2 involved feasibility testing of OHL-Act in a specialised diabetes centre. Results: Across implementing organisations, OS! was experienced as a practical approach supporting reflection and the generation of OHL improvement ideas, while also revealing barriers. These insights informed refinements, including clearer language, more structured facilitation guidance, and explicit prompts addressing health literacy challenges and high-risk situations. Feasibility findings indicated that OHL-Act could be delivered as intended and was perceived as acceptable, relevant, and useful in supporting reflection and the generation of OHL improvement ideas. Conclusions: OHL-Act represents a structured, co-creational approach to support OHL work. Further research is needed to examine how generated improvement ideas translate into sustained action and their potential implications for equity.

## 1. Introduction

Health systems worldwide are becoming increasingly complex, with expanding services, technologies, and administrative procedures, placing growing demands on people’s ability to navigate, understand, and use health information [1,2,3,4,5]. People with health literacy challenges often experience difficulties accessing services, communicating with professionals, understanding health information, and participating in decision-making [5,6,7]. These challenges are consistently associated with poorer care experiences and health outcomes [8,9,10,11] and disproportionately affect people in socially and economically unfavourable positions [5,12,13], thereby reinforcing health disparities. In response, attention has shifted from individual responsibility toward organisational accountability and the need to reduce system-level barriers that hinder equitable access to healthcare and meaningful participation in care [1,5].

Organisational health literacy (OHL) highlights organisations’ responsibility to make health information and services accessible, understandable, and usable for all [1,7]. Achieving OHL requires the implementation of strategies at multiple organisational levels, addressing not only direct interactions with service users but also the professional practices and organisational structures that shape these interactions [1,3,14]. Despite growing recognition of OHL as a key mechanism for promoting equity and improving quality of care [15,16], many healthcare organisations still lack the awareness, structures, and practical tools needed to systematically assess and embed OHL in everyday practice [17,18].

A number of tools and frameworks have been developed to support organisations in assessing and improving their OHL [18]. Among these is the OS! approach (hereafter OS!) [19,20], developed in Denmark through the adaptation of the Australian Organisational Health Literacy Responsiveness (Org-HLR) process and tools [21,22]. The Org-HLR was translated and iteratively adapted through pilot testing in three Danish healthcare centres, resulting in OS!. Unlike many existing tools [18,23], the Org-HLR and OS! perform the OHL assessment as a co-creative process engaging front-line staff and managers in identifying and prioritising locally relevant OHL improvement ideas [20]. Such participatory bottom-up processes may foster ownership and commitment and help initiate organisational changes required for the sustainable implementation of OHL improvements [3,17,18,24].

Detailed descriptions of OS! are available elsewhere [19,20]. Briefly, OS! retained the overall structure of the Org-HLR, scaffolded around three co-creational workshops. However, it replaced the original questionnaire-based self-assessment with open-ended questions and a more flexible, dialogue-based format [20]. Since 2019, OS! has been implemented across various Danish health organisations, often motivated by efforts to reduce health literacy-related disparities. However, to date, OS! has not been systematically evaluated. This leaves limited understanding of how co-creative OHL approaches are implemented and experienced in practice, and how such user-based insights can inform meaningful refinement.

Accordingly, this study aimed to: (1) examine the implementation experiences of OS! to inform refinements enhancing its relevance, engagement, and potential effectiveness; and (2) examine the feasibility of the refined version, named OHL-Act, in a healthcare setting.

## 2. Method

### 2.1. Design and Framework

In line with the two aims, the study was conducted in two sequential phases: a formative evaluation (Phase 1) and a feasibility study (Phase 2) of a complex intervention (i.e., OHL-Act). Data were collected between April 2024 and June 2025. The RE-AIM framework guided the study, providing a structured analytic lens across both phases [25,26]. Widely used to assess the implementation and impact of public health interventions [25,26,27,28], the five dimensions of RE-AIM—Reach, Effectiveness, Adoption, Implementation, and Maintenance [25,26]—informed both data collection and analysis.

**Phase 1** comprised a user-based formative evaluation of OS!, designed to generate practice-based insights to inform intervention refinement. This formative design aligns with guidance from the Medical Research Council (MRC), which emphasises early evaluation to optimise complex interventions prior to feasibility testing [28]. Formative evaluation is particularly relevant for co-creative approaches, where iterative and responsive adaptation is essential to maintain relevance and engagement [29].

**Figure 1:**
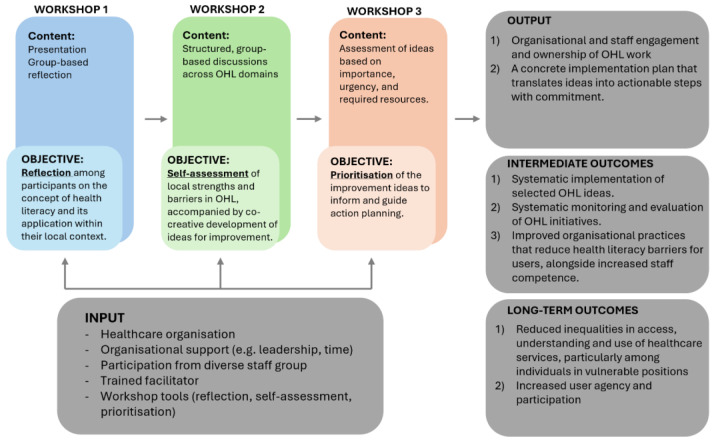
The core structure and objectives of OS! and OHL-Act, including inputs, workshop activities, outputs, and intermediate and long-term outcomes.

**Phase 2** comprised a feasibility study of the refined version, named OHL-Act. Consistent with guidance on feasibility studies for complex interventions [30,31,32], the study examined the practical deliverability and organisational acceptability of OHL-Act. It also examined at a general level participants’ perceptions of its usefulness in supporting reflection and the generation and prioritisation of improvement ideas. Across both phases, the core structure and objectives of the approach remained unchanged: a three-workshop, co-creational, bottom-up process designed to support organisational reflection, self-assessment, and prioritisation of OHL improvement ideas (Figure 1).

### 2.2. Phase 1: Formative Evaluation of OS!

#### 2.2.1. Data Collection and Participants

The Danish healthcare system is primarily publicly funded, providing universal access to healthcare services for all residents. Five regions are responsible for hospital services and collaborations with general practitioners, while the 98 municipalities manage health promotion, prevention, and rehabilitation [33]. At the time of the study, the research team was aware of nine Danish healthcare organisations that had implemented OS!, including one regional and eight municipal organisations. Participants were recruited using purposive sampling, i.e., based on their specific insight into OS! from these organisations or from teams responsible for implementing OS! within them [34].

To capture participants’ experiences of the OS!, semi-structured interviews were conducted. The interview sample comprised seven interviews, including one in-person and six online interviews due to geographical distance. Participants represented four different healthcare settings and held different roles in relation to OS!. The sample included three OS! facilitators, and four OS! participants (two front-line staff and two managers), with varying time since engagement with OS! (Table 1). The sampling focused on organisational roles and experience with OS! and the distribution regarding gender, age or professional background was not considered. However, the gender distribution (six women and one man) generally reflects the predominantly female composition of the Danish healthcare sector. In the OS! approach, facilitators are appointed by the implementing organisations. The research team had no influence on the selection of facilitators.

Interviews were guided by a semi-structured interview guide designed to elicit participants’ experiences with implementing OS!. The RE-AIM framework informed the interview guide [25,26]. *Adoption* was explored by asking why organisations chose to engage in OS! and how relevant participants perceived the approach to be. *Implementation* was examined by questions about experiences with the workshop format, facilitation, clarity of materials, and contextual adaptations. Questions related to *effectiveness* prompted participants to describe the types of improvement ideas generated, the perceived value of the process, and whether OS! influenced understanding of health literacy. Questions on *maintenance* focused on follow-up activities, barriers to sustaining OHL ideas, and how organisational structures supported or impeded longer-term change. In addition, some questions spanned more than one RE-AIM dimension, primarily to support the formative design by eliciting overall reflections and suggestions for improvement. The complete interview guide is available in Supplementary Table S1.

All interviews were conducted in Danish and lasted approximately 60 min. They were all audio-recorded, and transcribed verbatim. Interview quotes presented in the manuscript were translated from Danish into English by ASDR and reviewed by CKR to ensure conceptual equivalence.

#### 2.2.2. Data Analysis

The interview data were analysed using a deductive approach informed by the RE-AIM framework [25,26] with the five RE-AIM dimensions guiding the organisation of codes and themes. At the same time, the initial coding process remained open to data to allow for the identification of issues not captured by the framework. The analysis followed the three-step methodology described by Brinkmann [35].

1. Initial coding: Two authors (CKR and ASDR) independently read and re-read the transcripts to identify meaningful units of text, each expressing a distinct experience or reflection relevant to the study aim. Codes were generated separately through an iterative movement between raw data and emerging interpretations. This independent coding process was undertaken to enhance analytic rigour and reduce the influence of individual interpretive bias. Any discrepancies were resolved through discussion until consensus was reached.
2. Thematic organisation: Codes were compared, discussed, and organised within the five RE-AIM dimensions. The framework guided the analytic structure, while openness to data-driven nuances ensured that themes captured both shared patterns and variations across participants.
3. Meaning condensation: Each theme was further analysed through meaning condensation, revisiting the underlying coded units to identify their essential meaning and reformulate them into concise, analytically focused descriptions. This process involved iterative comparison between data, codes and emerging themes, reducing overlap and strengthening conceptual clarity. The resulting themes constitute the final thematic structure of the analysis. These themes are presented in Section 3 as implementation-related experiences, including perceived strengths and barriers of OS!.

#### 2.2.3. Reflexivity

CKR conducted all interviews and, together with ASDR, carried out the primary coding and analysis. Independent coding and subsequent analytic discussions between CKR and ASDR contributed to investigator triangulation and strengthened analytical credibility. Authors involved in the development of OS! (AA and HTM) did not participate in coding or primary data analysis, ensuring deliberate role separation during the analytic phase.

Neither CKR nor ASDR had been involved in the development of OS! prior to this study. To provide contextual insight into how OS! was implemented in practice, CKR observed two workshops at one organisation. The observations were exploratory and were not formally coded or included in the analytic dataset. Following completion of the analysis, the analytically derived themes and proposed refinements were discussed within the full research team to promote reflexive awareness and minimise the risk of confirmatory bias.

#### 2.2.4. Refinement

The analytically derived themes from Phase 1 were presented by CKR to the research team and discussed in depth. Based on these themes, CKR drafted proposed revisions to OS!. The proposals were iteratively discussed and critically reviewed by the full research team across several feedback rounds to enhance relevance, engagement, and the practical application of the approach. Consensus was ultimately reached on a set of changes, which were consolidated into OHL-Act.

### 2.3. Phase 2: Feasibility Study of OHL-Act

#### 2.3.1. Setting and Participants

The feasibility study was conducted at a large, specialised diabetes centre within the hospital sector in the Region of Southern Denmark. Managers at the centre approached the research team with an interest in strengthening OHL, and participation in the feasibility study of OHL-Act was subsequently agreed. 

In total, 25 staff members participated in Workshops 1 and 2. These participants represented a broad range of organisational roles, including managers, clinical leaders, healthcare professionals (nurses, physicians, dietitians), quality and education staff, and external collaborators from primary care. Workshop 3 involved 10 participants and primarily comprised senior management and selected clinical leaders.

Participant invitations were managed exclusively by the diabetes centre. In accordance with the OHL-Act facilitation guide, the research team recommended broad multidisciplinary representation and a majority of participants with direct clinical contact to ensure practice-based perspectives. These recommendations were only partially fulfilled, particularly in Workshop 3, which included a higher proportion of senior management than originally intended. The research team did not select which staff members were invited to participate.

#### 2.3.2. Delivery of OHL-Act

OHL-Act was delivered in accordance with the detailed facilitator guide developed in Phase 1 [36]. The facilitator guide allows flexibility in the organisation of exercises, provided that all core components are completed. The overall duration of the workshop programme remained consistent with the planned timeframe, and an experienced facilitator of OS! (AA) facilitated all OHL-Act workshops.

Workshops 1 and 2 were conducted consecutively as a single six-hour session in May 2025 rather than as two separate sessions of two and four hours. This adjustment reflected practical considerations within the clinical setting while maintaining the intended structure and core components of the intervention [36].

In Workshops 1 and 2, participants were divided into four groups. Educational staff from the diabetes centre facilitated discussions within each group and documented outputs using the structured exercise templates [36].

Workshop 3 was conducted in June 2025 as a four-hour session and delivered in accordance with the facilitator guide. The ten participants were divided into two groups to complete the prioritisation exercises outlined in the guide. The groups subsequently reconvened for the final prioritisation of improvement ideas.

#### 2.3.3. Feasibility Assessment

Feasibility was assessed using all five dimensions of the RE-AIM framework, with particular emphasis on adoption, implementation, and effectiveness. In this study, adoption was understood as organisational acceptability, implementation as practical deliverability, and effectiveness as participants perceived usefulness of OHL-Act in supporting reflection and the generation and prioritisation of improvement ideas.

To capture these dimensions, two structured questionnaires were developed and administered following completion of the workshops. The questionnaires included both closed- and open-ended items. Closed-ended items used a five-point Likert scale ranging from “not at all” to “to a very high extent”. The questionnaires are available in full in Supplementary Tables S2 and S3.

Reach was examined in relation to staff participation, as OHL-Act targeted organisational actors rather than service users. Maintenance was assessed only through short-term, intention-based indicators, including participants’ intentions to continue working with OHL and their perceptions of organisational commitment to follow-up activities, rather than through long-term outcome measures.

Finally, questionnaire items were informed by analytically derived themes from Phase 1 that had guided the refinement of OHL-Act. This allowed the feasibility assessment to examine how the refined elements were experienced.

The first questionnaire was piloted among four healthcare professionals and researchers with expertise in health literacy. The second questionnaire was piloted internally within the research team. Both pilot processes resulted in minor adjustments to improve clarity and wording. The questionnaires were administered immediately after Workshops 2 and 3 by CKR.

In addition to the questionnaire data, exploratory observational notes were documented during the workshops to capture group processes, interaction patterns, and practical delivery considerations. These observations served to contextualise the feasibility assessment but were not formally included as part of the quantitative analysis.

Closed-ended questionnaire data were analysed descriptively to summarise response distributions across items. Responses to open-ended items were limited and were reviewed qualitatively by CKR to identify comments relevant to practical deliverability, organisational acceptability, and perceived usefulness. Given the exploratory purpose of this early-stage feasibility study, the analysis aimed only to provide indicative insights rather than statistical inference.

#### 2.3.4. Final Adjustments

Combined findings from the questionnaires and workshop observations were reviewed by the research team and resulted in minor refinements aimed at enhancing linguistic clarity and practical deliverability in the final version of OHL-Act [36]. 

An overview of the final version is provided in Supplementary Table S4, and a detailed description is provided in Supplementary Table S5. An English-language version of OHL-Act is currently under preparation and will be made publicly available.

### 2.4. Ethics

According to Danish legislation, ethical approval is not required for non-invasive health research. The study was approved by the Danish Data Protection Agency (journal no. 2022-0367531, ref. 3563). Written informed consent was obtained prior to interviews and questionnaire completion. 

Beyond formal approval procedures, ethical considerations included attention to relational dynamics and the influence of researcher positionality on access, dialogue, and interpretation. Ongoing reflexive awareness was maintained to support trust and responsible conduct throughout the research process.

## 3. Results

### 3.1. Phase 1: Formative Evaluation of OS!

The analysis identified both strengths and barriers related to the implementation of OS!. Findings are presented thematically and structured according to the RE-AIM framework, reflecting the analytic approach. Illustrative quotes are provided in Table 2 and Table 3, and the key refinements informed by these findings are summarised in Table 4.

#### 3.1.1. Perceived Strengths Related to the Implementation of OS!

The analysis indicated that participants experienced OS! as adaptable across diverse healthcare settings and capable of engaging managers and staff at multiple organisational levels. Participants described the workshops as fostering collective ownership, meaningful dialogue, and a shared understanding of health literacy, while prompting professional reflection on everyday practices.

The practical, dialogue-based workshop format was perceived as supporting the generation of improvement ideas and locally anchored action plans. Context-specific adaptations were described as supporting implementation. Engaged leadership with clearly distributed responsibilities was considered important for maintaining momentum and following up on selected improvement initiatives (Table 2).

#### 3.1.2. Perceived Barriers Related to the Implementation of OS!

Participants described that implementation of OS! was, in some settings, challenged by staff fatigue related to earlier unproductive processes and limited local influence on decision-making. A lack of clear leadership backing and conflicting organisational priorities were further perceived as constraining implementation in some settings. The approach’s dependence on skilled facilitation, the conceptual complexity of OHL, and the abstract nature of certain tools made it difficult for some participants to remain engaged during workshops. The self-assessment scoring system used in Workshop 2 was considered impractical by several participants. 

In some settings, the resource demands associated with OS! were perceived as high, and limited end-user involvement (i.e., patients and service users) was identified as a potential limitation. Sustaining change following the workshops was described as a challenge due to extended implementation timelines, staff turnover, resource constraints, system-level constraints and insufficient leadership support (Table 3).

#### 3.1.3. Refinements Informed by the Formative Evaluation

Findings from Phase 1 informed a series of targeted refinements addressing identified barriers and strengthening clarity, usability, and perceived relevance of the approach. These refinements resulted in the revised version, OHL-Act. The major refinements are summarised in Table 4.

In addition to data-driven refinements, the refinement process included analytic considerations by the research team. During this process, it was identified that equity-related considerations were not explicitly addressed within the existing self-assessment structure. To strengthen the systematic focus on disadvantage groups and local equity risks, a new domain titled “Vulnerability & High Risk” was introduced. This domain was designed to prompt structured reflection on health literacy-related challenges among users and on local organisational routines requiring high levels of health literacy.

### 3.2. Phase 2: Feasibility Study of OHL-Act

Of the 25 participants in Workshops 1 and 2, 21 attended the full session and completed the questionnaire. Four participants were unable to remain for the entire workshop due to clinical obligations and illness. All remining participants answered the 11 closed-ended items (100%, 21/21), while responses for the three open-ended items ranged from 6 to 11 responses out of 21. 

In Workshop 3, all 10 participants completed the workshop and questionnaire. Eight closed-ended items were answered by all participants (100% 10/10). Two remaining closed-ended items related to maintenance were completed by eight participants (80% 8/10), and the single open-ended item was completed by three participants (30% 3/10). Full distributions of questionnaire responses are presented in Supplementary Figures S1 and S2.

Across discussion groups in Workshop 2, 89 improvement ideas were generated. The ideas were reviewed and consolidated by merging overlapping proposals and excluding suggestions that were not OHL-related or addressed external conditions beyond the organisation’s control. Following this screening process, 44 ideas (49%) were carried forward to Workshop 3 for prioritisation (Table 5). The newly added “Vulnerability & high risk” domain generated the most ideas *(n* = 19). Several of these overlapped, and two were ultimately prioritised in Workshop 3 (Table 5).

During Workshop 3, 22 ideas were prioritised independently by the participants for implementation based on perceived importance and feasibility. The prioritised ideas spanned user-, professional- and organisational-level actions.

#### 3.2.1. Reach

Workshops 1 and 2 included representation across professional roles within the organisation. Workshop 3 primarily involved senior management and selected clinical leaders, reflecting a narrower representation during the prioritisation phase. In addition, six out of ten respondents indicated that certain staff groups were not fully represented in the process, most frequently front-line clinicians.

#### 3.2.2. Effectiveness (Perceived Usefulness)

Participants reported that OHL-Act supported reflection of OHL and facilitated the generation of concrete OHL improvement ideas. In Workshops 1 and 2, 61.9% (13/21) indicated that they gained new insights into OHL “to a high” or “very high extent,” and 71.4% (15/21) reported that the workshop helped generate useful ideas. In Workshop 3, all participants indicated that the session contributed to agreement on which initiatives to prioritise for implementation.

#### 3.2.3. Adoption (Organisational Acceptability)

Participants across professional roles reported that OHL-Act was relevant to their organisational context and aligned with existing priorities. In Workshops 1 and 2, 85.7% (18/21) indicated that OHL aligned with current initiatives “to some extent”, “to a high extent” or “to a very high extent”. Observations and feedback from inhouse group facilitators during Workshops 1 and 2 indicated active participation in discussions and group work including consistent participation from senior managers throughout the sessions. This suggests that OHL-Act was regarded as acceptable in the organisational context, supporting adoption. In Workshop 3, all respondents rated participation as important “to a high” or “very high extent”.

#### 3.2.4. Implementation (Practical Deliverability)

Participants generally rated the OHL-Act materials and questions as clear and understandable, although some noted that certain questions discussed in Workshop 2 were complex. Across Workshops 1 and 2, 95.2% (20/21) reported that the questions provided a good basis for discussion “to some”, “high”, or “very high extent”.

Engagement was reported as high, with 90.5% (19/21) indicating active participation in group discussions. Observations and open-ended responses indicated that the combined duration of Workshops 1 and 2 was experienced as intensive.

The red–yellow–green rating format introduced in Workshop 2 to replace the earlier numerical scale, recieved mixed responses. Although it was intended to simplify scoring and facilitate dialogue, 47.6% (10/21) found it useful only “to a limited extent” or “not at all,”. These findings were supplemented by observations indicating that the rating system, sometimes distracted participants from engaging with the substantive discussion points. This led to the scales’ removal in the final version of OHL-Act.

#### 3.2.5. Maintenance (Short-Term Indicators)

Short-term indicators of maintenance were identified following Workshop 3. All participants indicated willingness to continue contributing to OHL-related work. During the final part of the workshop, senior managers discussed potential mechanism to support ongoing progress. Although a formal action plan has not yet been finalised, a responsible lead has been appointed, and development of the plan is underway, accompanied by strategic decisions to enable OHL improvement initiatives and share learning with relevant partners.

## 4. Discussion

### 4.1. Key Findings

This study conducted a formative evaluation of OS! and developed a refined version, OHL-Act, based on identified strengths and barriers. Across implementing organisations, participants described OS! as a practical approach that supported reflection and the generation of OHL improvement ideas (Table 2). However, participants highlighted conceptual complexity, facilitation demands, and resource requirements as practical challenges in implementing. Strong leadership and clear distribution of roles and responsibilities were described as important conditions for sustaining implementation efforts (Table 3). Limited explicit focus on disadvantage groups within the self-assessment structure was identified during the refinement process, leading to the introduction of a dedicated “Vulnerability & High Risk” domain in OHL-Act (Table 4).

While a direct comparison with OS! was not possible, feasibility data indicated that OHL-Act was perceived as relevant, linguistically clear, and easy to participate in, supporting its practical deliverability and acceptability. The workshops generated engagement around OHL initiatives and a set of locally anchored improvement ideas, including initiatives addressing equity-related aspects of OHL (Table 5). Although time constraints and the lack of end-user involvement remained barriers, respondents expressed willingness to continue working with OHL and identified next steps for further development.

### 4.2. Interpretations

The refinement of OS! into OHL-Act was informed by barriers identified during the formative evaluation. In particular, the conceptual complexity of OHL and the difficulty of translating theoretical OHL principles into concrete, context-appropriate ideas for organisational improvement emerged as a central barrier. This aligns with broader critiques indicating that, despite their conceptual richness, many OHL tools provide limited practical guidance necessary for application in healthcare settings [3,18,23]. In response, linguistic and structural simplifications were introduced in OHL-Act, including fewer vaguely defined terms, more explicit prompts, and concrete examples. These refinements were indented to reduce cognitive and interpretive demands to support more focused and actionable discussions of OHL in practice. However, the implementation of OHL-Act remains dependent on organisational conditions that shape participants’ motivation, sense of purpose and openness in the process. Consistent with the initial testing of OS! [20] and field-testing of the Australian Org-HLR tool [38], the identification and prioritisation of improvement ideas was supported by careful facilitation, strong leadership support, and engaged participation across organisational levels.

The limited explicit focus on identifying and reflecting on high-risk situations and target groups in OS! informed the refinement of OHL-Act, where this dimension was made more structurally visible. Although OS! was often implemented with the intention of supporting equity, the original materials primarily addressed this aim indirectly through general OHL principles.

Equity is widely recognised as a central rationale for OHL [1,18]. However, evidence linking OHL initiatives to measurable equity improvements remains limited [3,17,18], and equity considerations are often insufficiently operationalised in existing OHL guides and tools [18,23]. Building on the well-documented social gradient in health literacy, Nutbeam and Lloyd (2021) argue that health literacy interventions should adopt a proportionate universalism approach, whereby *“priority should be proportionate to need, with particular attention given to reaching and engaging population groups who are disproportionately affected by low health literacy”* [5]. From an organisational perspective, this implies combining universal system-level approaches with targeted attention to contexts in which health literacy demands are particularly high. In the feasibility study, the addition of a domain focusing on high-risk situations and target groups appeared to engage participants in explicit equity-related reflections, as reflected in the generation of improvement ideas within this domain. The presentation of national recommendations framing OHL as a strategy to enhance equity [37] may also have contributed to participants’ attention to equity-related considerations. Although the national recommendations are published for a Danish audience, they align closely with widely acknowledged OHL frameworks [1,18]. The underlying approach—explicitly foregrounding equity considerations in the presentation of OHL—may therefore be transferable beyond the Danish context. Whether such explicit foregrounding of equity within organisational reflection processes translates into measurable equity outcomes remains to be investigated.

End-users are generally not directly involved in OS! or OHL-Act workshops. Both approaches primarily target organisational systems and professional practices, consistent with other OHL tools [1,4,23]. While user involvement is included as a domain within OHL-Act and framed as an important component of OHL development, participation of end-users in the workshops has not been systematically integrated into the intervention format. End-user participation is commonly included in public health interventions based on co-creation [24,39], and its limited direct integration in OHL approaches may represent a structural tension between systems-level change and participatory ideals. Further research is needed to explore how end-user perspectives can be meaningfully integrated into OHL initiatives, including strategies to ensure adequate representation of individuals experiencing health literacy challenges [40].

From a maintenance perspective, findings from the formative evaluation indicate that sustaining momentum after OS! is closely linked to a clear allocation of responsibility, staff engagement, and leadership support. Organisations lacking these conditions appeared to experience greater difficulty in translating ideas into practice. These findings align with organisational change literature identifying leadership support as a key facilitator of sustained change [38,41,42], as well as with co-creation research highlighting the importance of ownership for continued action [24,43]. By involving local staff in generating and prioritising improvement ideas, OHL-Act is designed to foster ownership and local relevance, which may support subsequent implementation efforts [24,43,44]. However, the implementation of prioritised improvement ideas and their long-term sustainability were conceptualised as downstream outcomes of OHL-Act (Figure 1) and were therefore beyond the scope of this feasibility study.

Evidence on sustaining OHL development remains limited. The concept of *“health literacy champions”* has been identified as one potentially promising strategy for supporting continued action over time [5,45]. Accordingly, OHL-Act recommends that prioritised OHL initiatives be accompanied by the appointment of a responsible individual or group to support follow-up [46,47]. Further research is needed to examine the competencies, roles, and contextual conditions that enable effective health literacy champions, as well as the mechanisms through which OHL initiatives influence organisational practices and their longer-term implications for health equity.

### 4.3. Strengths and Limitations

A key strength of this study was the consistent application of the RE-AIM framework, which provided a structured and theory-informed lens for both the formative evaluation of OS! and the feasibility study of OHL-Act. This approach ensured a systematic focus on organisational conditions related to implementation, adoption, maintenance, and perceived usefulness in practice. The use of a shared framework across phases strengthened conceptual coherence and facilitated transparent reporting of both evaluation and feasibility findings.

More broadly, both OS! and OHL-Act were evaluated through empirical application. This addresses an important gap in the OHL field, where many tools are conceptually developed but less frequently examined in practice [3,18].

Access to former OS! users through the developers (AA and HTM) enabled recruitment of participants with direct experience of the approach. However, this recruitment pathway may have increased the likelihood that participants emphasise positive experiences. To mitigate this risk, all interviews were conducted by CKR, who was not involved in the development of OS!, and reflexive awareness of relational dynamics was maintained throughout the interview process.

Questionnaires were distributed immediately after the workshops to maximise response rates. While this ensured high completion, the timing may have influenced reported engagement and satisfaction despite anonymous responses.

The formative evaluation included a small number of participants (*n* = 7), which may have limited the diversity of perspectives captured. However, several themes recurred across interviews, suggesting a degree of consistency in participants’ experiences. While participants represented variation in organisational roles and settings, sampling did not consider other factors of representativeness. The interview sample was predominantly female, reflecting the gender composition of the Danish healthcare sector. Gender was not an analytical focus of the study, but Phase 1 findings should of cause be interpreted with the purposive sampling strategy in mind.

For some participants, a considerable time had passed since their participation in OS!, which may have affected recall of workshop details but allowed for reflection on longer-term organisational processes, including maintenance. End-users were not involved, limiting insight into their views on OHL-related changes and constrained conclusions regarding reach.

Additionally, issues of researcher positionality should be acknowledged. Familiarity with the organisational context, supported by preliminary workshop observations, strengthened the interpretation of implementation processes and underlying assumptions. However, such contextual closeness may also have entailed a risk of taken-for-granted understandings or selective attention to themes resonating with the researchers’ professional background. Although reflexive dialogue and analytic triangulation were applied to mitigate this risk, some degree of interpretive influence cannot be entirely excluded.

The feasibility study demonstrated active generation of locally anchored OHL improvement ideas in Workshop 2. Many ideas targeted practices and policies at a relatively high organisational level [1,14], which contrasts with patterns observed in some previous OS! processes. This may reflect the composition of participants, several of whom held managerial responsibilities and were therefore accustomed to organisational-level thinking, suggesting that participant composition influences the level of ideas generated.

Workshop 3 primarily involved senior management, which may have further shaped prioritisation dynamics and the perceived relevance of selected initiatives across professional levels. At the same time, leadership involvement in the prioritisation phase may facilitate strategic alignment and organisational anchoring. Future applications of OHL-Act should therefore consider balancing managerial and front-line representation to optimise reach and shared ownership [1,14]. Finally, the feasibility study was conducted in a single, highly specialised diabetes centre and primarily involved managerial staff, which may limit transferability to other healthcare settings and participant compositions. Moreover, as members of the authoring research team facilitated all workshops, conclusions regarding the usefulness of the facilitator guide for less experienced facilitators are still to be made. Nevertheless, the formative evaluation and feasibility findings suggest that the refined and more concrete workshop materials, may support application in another organisational context. Further testing in diverse healthcare settings is needed to assess broader applicability.

### 4.4. Implications and Future Directions

The findings suggest that OHL-Act may serve as a structured approach to support organisational reflection and prioritisation of OHL initiatives in healthcare settings. The feasibility study indicates that explicit prompts and a clear workshop format can facilitate locally anchored idea generation. However, participant composition appears to influence the level and type of initiatives generated and prioritised, underscoring the importance of clarifying local aims when planning implementation. At the same time, effective use of the approach appears to depend on organisational readiness, including leadership support, facilitation capacity, balanced participant representation, and clear allocation of responsibility for follow-up.

With regard to equity, OHL-Act introduces explicit prompts related to health literacy challenges and high-risk situations. By foregrounding these considerations within structured reflection processes, the approach may encourage organisational attention to equity-related issues, even when end-users are not directly involved.

Future research should examine how improvement ideas generated through OHL-Act are translated into sustained organisational actions over time, and how contextual factors facilitate or hinder this process. Further studies are also needed to explore potential equity-related implications and to assess implementation of the facilitator guide when used independently of the developing research team.

## 5. Conclusions

This study provides practice-based insight into how a co-creational, bottom-up workshop format can support organisational reflection on OHL and the development of locally relevant improvement ideas.

The formative evaluation of the original OS! based on purposive sampling and qualitative information analysis identified areas requiring refinement to enhance clarity, engagement, and feasibility, highlighting the value of practice-based evaluation of OHL tools in healthcare settings.

The feasibility study of OHL-Act indicated that the refined approach could be delivered as intended in a highly specialised healthcare setting and was perceived as acceptable across organisational levels. The findings suggest that OHL-Act may represents an initial step in supporting structured reflection, idea generation, and prioritisation related to OHL. Further research is needed to examine how improvement ideas generated through OHL-Act are translated into sustained organisational action and how such initiatives may influence equity over time.

## Figures and Tables

**Table 1:** Characteristics of participants included in the Phase 1 formative evaluation.

ID	Gender	Healthcare Setting/Organisational Setting	Role in Relation to OS!	Approximate Time Since Engagement
ID A	Female	Municipal health centre—Prevention unit	Facilitator	<6 months
ID B	Female	Regional hospital setting	Facilitator	>12 months
ID C	Female	Municipal health centre—Prevention unit	Participant (front line staff)	<6 months
ID D	Female	Regional hospital setting	Participant (front line staff)	>12 months
ID E	Female	Municipal health centre—Health promotion and prevention services	Participant (manager)	>24 months
ID F	Male	Municipal rehabilitation service	Decision maker (manager)	>12 months
ID G	Female	Municipal health centre—Health promotion and prevention services	Facilitator	>24 months

**Table 2:** Strengths of OS! as perceived by participants.

Strength	Description	Example Quote *	RE-AIM
Broad organisational reach with wide staff involvement	The OS! was perceived as adaptable across different types and sizes of healthcare organisations. The approach enabled staff from across the organisation to engage in the process, thereby fostering collective ownership and meaningful discussions.	ID F: It really must involve everyone working with differentiated approaches. It simply doesn’t work if the person sweeping outside says to someone who can barely manage to get off their tricycle and their way back while holding onto the wall, that “You can’t leave your bike there”. Or if, when they reach the counter, there is no one able to receive them, even though they had spoken on the phone beforehand and been told: “Just come up, we’ll take care of you”.	Reach
Shared understanding and reflective practice	The OS! contributed to a shared language about health literacy and sparked professional reflection.	ID C: What has really worked well is that we have developed a more shared language around the concept of health literacy. It has also become an integrated part of our work. We got a new Electronic Health Record, where it is actually something we are required to address… We have gained a common language for some of the things we may have talked about before.	Effectiveness
Numerous improvement ideas and context-specific action planning	The OS! led to the development of action plans and numerous improvement ideas anchored in local organisational practice.	ID G: I think it’s absolutely crucial. That there is a concrete action plan, because that is also what ensures proper follow-up.	Effectiveness
Practical workshop approach fostering comprehensive organisational reflection	The OS! was valued for its practical, workshop-based format that enabled organisations to critically reflect on their own practices. As a tangible approach, it offers a comprehensive 360-degree perspective on organisational health literacy.	ID G: Organizational health literacy can indeed be a very large and abstract concept but having a tool to talk about it is really helpful in terms of moving from this abstract level to something more concrete. It provides good opportunities to adapt and develop concrete ideas about what we can do to improve organizational health literacy in our own setting.	Adoption
Organisational motivation from internal curiosity and external attention	Organisations were motivated to undertake the OS! by curiosity about their own structures and the populations they serve, as well as the anticipated benefits for organisational health literacy. Growing national attention to health literacy increased its relevance.	ID C: So, it was also kind of a self-check for us, to see whether we are actually good enough to reach this target group, namely the most challenged citizens—some of those with low health literacy. They care the ones where there is the most health to gain, so to speak, and are we actually good enough to accommodate the issues they bring? (…) We also have a manager who has the perspective that it’s okay for us to do self-checks.	Adoption
Staff motivation driven by involvement, relevance, solutions, and leadership support	Staff motivation was strengthened by involvement and discussing two of the six domains “access” and “communication”, which were seen as directly relevant to practice. Clinicians showed a solution-focused mindset, supporting the OS!’s objective of generating improvement ideas. Strong leadership provided clear strategic support for organisational health literacy as a shared priority.	ID B: And that thing about communication. It is tangible, and of course it’s on everyone’s mind, but it’s also perhaps one of the easiest things to relate to.	Adoption
Adaptable approach with interactive and text-level adjustments tailored to organisational context	Implementation of the OS! involved multiple context-specific adaptations, including interactive workshop adjustments, alignment with national health literacy standards, and text refinements for clarity and usability.	ID A: We prepared some other questions based on the questions from the reflection workshop in the OS! but adjusted them so that we felt they were a bit more relevant for our context.	Implementation
Strong leadership and shared ownership sustain ongoing change	Clear distribution of responsibilities and strong leadership support were key to maintaining momentum and ownership after OHL-Act.	ID E: Everyone is part of some working groups. Some are part of a few more, but it is to distribute responsibility (…) So some things move faster and others slower. Otherwise, we bring it up at our staff meetings. And twice a year, we go through this entire scheme, also to talk a bit about the things we have actually implemented.	Maintenance

* All quotations were originally in Danish and have been translated into English by the authors.

**Table 3:** Barriers to OS! as perceived by participants.

Barrier	Description	Example Quote *	RE-AIM
Lack of end-user involvement	End-user involvement was absent in most organisations. When included, participation was challenging, sometimes inhibiting clinician discussion	ID B: Well, I think this thing about the citizen, patient, and user is also somewhat absent in a way, and it’s not that easy (…) We had some citizens participate in workshop 1, and it wasn’t particularly easy for them either. Something also happens for the clinicians when citizens are present. There are some things you might be more hesitant about. Honestly, I also think it’s difficult for the citizens to relate to, so no, I don’t think it worked the way it’s structured now [OS!] (…) Of course, it then requires more resources for a process facilitator.	Reach
Insufficiently concrete improvement ideas	Many ideas were perceived as insufficiently concrete and required significant follow-up work by the facilitator	ID A: And when you get to the prioritization workshop and are supposed to prioritize actions, not everything has actions attached to it. I mean, a lot of issues have been generated or discussed, but no action proposals have been made. And then there’s nothing to prioritize.	Effectiveness
Staff resistance due to fatigue and limited influence	Repeated processes without visible results prior to undertaking the OS! led to staff fatigue and frustration. A perceived lack of influence in decision-making further lowered motivation for the approach, while inconsistent attendance and low prioritisation of workshop participation hindered engagement.	ID D: That feeling of resignation or that somewhat ’no’ attitude, because we have… We have tried this before. It doesn’t work, or it doesn’t help at all, because of management or, you know, all those different things.	Adoption
Challenges related to leadership support and priorities	Lack of clear leadership support and underlying conflicting priorities posed challenges for the OS! implementation. Support and engagement at the leadership level were perceived to require a “burning platform”.	ID G: They (the managers) are almost always represented in some way on the day, but many of them are also involved in so many things that many of them have to run back and forth.	Implementation
Facilitator role demands experience and faces multiple challenges	The OS! is highly dependent on skilled facilitation, requiring substantial experience for successful implementation. In some organisations, the approach benefited from the capacity of an external facilitator. Various challenges arose during facilitation, partly due to the facilitation guide’s high level of abstraction, which at times complicated practical application.	ID G: But I also think it requires a lot of facilitation to help them [OS! participants] be specific. Because I also experience that it’s really difficult for them be specific. What they are discussing can easily become very abstract. Just in general.	Implementation
Complexity and conceptual challenges in implementation	The concepts of health literacy and organisational health literacy required substantial introduction, and the comprehensive nature of the approach made it difficult for participants to stay focused and concrete during workshops. Some domains, particularly “leadership and systems,” were perceived as highly abstract, complicating engagement.	ID B: That there were some of the questions that were too difficult for them [participants], or that did not make sense for them to respond to.	Implementation
High resource requirements	Undertaking an OS! approach requires substantial resources, including facilitator planning and significant staff time away from daily routines to participate in and prioritise workshops.	ID B: It [OS!] is very broad. And at the same time, I would also say it’s relatively complex, because it’s so large. It [OS!] is not just like bang bang bang. It’s also quite demanding workshops.	Implementation
Limitations of the OHL-Act self-assessment scoring system in Workshop 2	The self-assessment scoring system in Workshop 2 was designed for longitudinal evaluation but has not been used this way by any organisations known to the participants. Instead, it primarily served as a dialogue tool. The 0–4 scale was seen as difficult to apply consistently and somewhat irrelevant, taking up valuable time that participants already found limited.	ID F: And of course, some of us also really like those quirky numbers and thought it was a bit fun to get a score out of it, but I can see now, that because of A, B and C, we haven’t had time to repeat it (OS!)	Implementation
Organisational barriers to implementing improvement ideas following the OHL-Act approach	Sustaining organisational changes proved challenging due to longer-than-expected timeframes and frequent staff turnover. System-level changes were difficult to implement, while political and bureaucratic constraints often hindered progress. Moreover, insufficient leadership support complicated efforts to maintain momentum and implement improvement ideas generated during the workshops.	ID E: But that’s also because certain things happen. Then we still run into sick leaves. And then we have a temp that we hired, who is now pregnant, and these are the kinds of things that happen in our everyday work. And we need to focus on the persons we are actually here for (…) So the entire implementation, carrying out all these things, has taken significantly longer than we ever imagined, but it’s small, small steps and all in all it’s going really well.	Maintenance

* All quotations were originally in Danish and have been translated into English by the authors.

**Table 4:** Major refinements from OS! to OHL-Act and their underlying rationale.

Component/Workshop	Refinement	Rationale
**General refinements**		
Naming and communication	OS! renamed to OHL-Act	The refined version was renamed to more clearly reflect its focus on OHL action and improvement.
Facilitator guide *	Comprehensive, practice-oriented facilitator guide developed with detailed pre-, during- and post-workshop instructions	To enhance feasibility, support standardised delivery, and provide structured guidance that may assist facilitators with varying levels of experience.
**Workshop 1—reflection**		
Revised reflection tool	The five discussion questions were rewritten to be concrete and practice-oriented	The formative evaluation found that the original questions were too complex
**Workshop 2—Self-assessment**		
Expanding from six to eight domains **	Added “Monitoring & Evaluation” and “Vulnerability & High Risk” domains	Overall, the eight-domain structure aligned OHL-Act with the Danish national recommendations on OHL, which prioritises system-level change to reduce health inequities with specific attention to people in high risk of health literacy challenges [37]. The recommendations are aligned with acknowledged OHL frameworks [1,18]. In the formative evaluation, equity considerations were perceived to be insufficiently captured in the self-assessment, prompting the addition of the ‘Vulnerability & High Risk’ domain to ensure that reflections regarding local risks of sustaining or worsening equity was addressed explicitly rather than implicitly. The domain focus on the identification of health literacy related challenges among users and local situations or routines requiring high level of health literacy. ‘Monitoring & Evaluation’ was separated into an independent domain to reinforce the need for data on local health literacy challenges and systematic follow-up.
Increase from 18 to 20 discussion questions	Expanding questions across the 8 domains	To retain the conceptual scope of OHL while stimulating reflection yet reduce the complexity and broadness of individual questions.
Refined wording and examples in for all discussion questions	Clearer phrasing, more concrete examples	To improve linguistic clarity and practical relevance, thereby supporting more constructive and innovative dialogue
Refined domain distribution	Max. four domains per group; ≥ two groups per domain	To ensure engagement and multiple perspectives per domain but reduce the burden on each participant as former participants experienced fatigue when discussing all domains
Removal of 0–4 scoring	Replaced with red–yellow–green dialogue tool	To promote dialogue rather than precise scoring, as participants often spent time debating small numerical differences instead of discussing improvement ideas. Although originally intended for longitudinal evaluation, the scale had not been used this way in practice and mainly functioned as a consensus tool. However, it was removed following the feasibility study.
**Workshop 3—Prioritisation**		
Added two alternative workshop formats for inspiration	Format suggestions for conducting Workshop 3 were added including expanded detailed instructions.	Workshop 3 does not have a fixed format. The refinement was added to support facilitators in selecting a format that fits their organisational context and to reduce uncertainties in planning and facilitating the workshop

*OHL = Organisational health literacy.* * The feasibility of the facilitator guide was not evaluated in this study. ** The final eight domains align with the Danish national recommendations on eight pathways to equity, framing organisational health literacy as an equity strategy [37].

**Table 5:** Number of improvement ideas generated across the eight OHL-Act domains at the diabetes centre during Workshop 2, merged and subsequently prioritised during Workshop 3, with illustrative examples.

Domain in OHL-Act	Total Number of Ideas from Workshop 2	Final Number *	Prioritised Ideas at Workshop 3 **	Examples of OHL Improvement Ideas
**Leadership & Culture**	9	4	3	Develop, implement, and communicate a comprehensive management strategy for organisational health literacy at the diabetes centre. The strategy should include a clear set of values, well-defined priorities for systemic and relational approaches, and a plan for differentiating services for patients with varying needs.
**Competencies**	9	6	3	All staff are offered training in health literacy as part of professional development, including the aim of fostering a shared language.
**Process & Practice**	11	5	4	Develop a differentiated model for services—for example, in the form of a large, medium, and small package according to different user-needs.
**Involvement**	12	5	3	Develop a plan to increase involvement of relevant user groups, with particular focus on vulnerable or hard-to-reach populations, for example by creating a broader user panel rather than relying on a selected group.
**Access**	11	7	4	Introduce more flexible scheduling by offering need-based appointment times across all staff, extending physician slots for patients with special needs, and providing select walk-in consultations.
**Communication**	13	4	2	Develop a range of communication materials supporting different learning styles and health literacy levels. Tailor verbal and written communication to the user needs, adjusting language, readability, and visual aids.
**Vulnerability & High Risk**	19	9	2	Establish structured cross-sector collaborations by appointing key coordinators (e.g., social nurse) to link hospital, municipality, and primary care, clarifying responsibilities, sharing knowledge, and creating joint workflows to support patients, especially vulnerable ones, during transitions.
**Monitoring & Evaluation**	5	4	2	Develop a systematic approach for feedback on interventions—including feedback from patients to clinicians.
**Total**	**89**	**44**	**22**	

* The reduction of ideas reflects merging of overlapping ideas and, in a few cases, the exclusion of suggestions that did not relate to OHL or fell outside the organisation’s remit. ** During Workshop 3, participants prioritised 22 of the 44 final ideas based on perceived importance and resource demands and categorising them as either feasible for short-term implementation (<6 months) or requiring longer-term implementation (>6 months).

## Data Availability

The data presented in this study are not publicly available due to ethical restrictions and concerns regarding participant confidentiality. Anonymised data may be made available from the corresponding author upon reasonable request and subject to ethical approval.

## Supplementary Materials

The following supporting information can be downloaded at: https://www.mdpi.com/article/10.3390/ijerph23030391/s1, Figure S1: Response frequencies (%) for feasibility-related items from workshops 1 and 2 (N = 21); Figure S2: Response frequencies (%) for feasibility-related items from workshop 3 (N = 10); Table S1: Semi-structured interview guide mapped to RE-AIM dimensions; Table S2: Overview of questionnaire items from workshops 1 and 2 used in the feasibility study, mapped to RE-AIM dimensions; Table S3: Overview of questionnaire items from workshops 3 used in the feasibility study, mapped to RE-AIM dimensions; Table S4: Overview of OHL-Act; Table S5: Detailed description of OHL-Act [36].
