# Supplementary material for: Co-Creating Organisational Health Literacy: Formative Evaluation and Feasibility Testing of OHL-Act
Source: Int J Environ Res Public Health. 2026 Mar 18;23(3):391. doi: 10.3390/ijerph23030391 (PMC13027036; doi:10.3390/ijerph23030391)
Supplement: Supplementary file 1 [file ijerph-23-00391-s001.zip › ijerph-4087194-Supplementary Materials.pdf]

International Journal of Environmental Research and Public Health Special Issue: Health Disparities and Health Literacy: Bridging the Gap

Supplementary file

Co-creating organisational health literacy: Formative evaluation and feasibility testing of the OHL-Act

Camilla Klinge Renneberg*[1], ORCID: 0000-0002-8588-7637

Anne Sofie Dydensborg Rasmussen[1], ORCID: 0009-0006-6422-5809

Maiken Meldgaard[1], ORCID: 0000-0003-4122-1793

Helle Terkildsen Maindal[1,2], ORCID: 0000-0003-0525-7254

Anna Aaby[1], ORCID: 0000-0002-1446-643X

Affiliations:

1. Department of Public Health - Aarhus University, Denmark
2. Institute for Health Transformation, Deakin University, Australia

*Corresponding author: Camilla Klinge Renneberg

Department of Public Health, Aarhus University, Bartholins Allé 2, 8000 Aarhus C, Denmark.

Supplementary table of contents

Page

Table S1: 3-4
Semi-structured interview guide mapped to RE-AIM dimensions

Table S2: 5
Overview of questionnaire items from workshops 1 and 2 used in the feasibility study, mapped to RE-AIM dimensions

Table S3: 6
Overview of questionnaire items from workshops 3 used in the feasibility study, mapped to RE-AIM dimensions

Table S4: 7
Overview of OHL-Act

Table S5: 8-11
Detailed description of OHL-Act

Figure S1: 12
Response frequencies (%) for feasibility-related items from workshops 1 and 2 (N = 21)

Figure S2: 13
Response frequencies (%) for feasibility-related items from workshop 3 (N = 10)

Table S1: Semi-structured interview guide mapped to RE-AIM dimensions

The guide served as a flexible support, allowing follow-up questions depending on participants' experiences and roles.

Interview questions	RE-AIM dimension
Can you briefly tell me about yourself, and your role or function in the organisation?	**Context**
Can you describe your workplace (e.g. size, professional groups, organisational structure, and main services or activities)?	**Reach**
Can you describe the target group(s) your organisation primarily supports?	**Reach**
Who participated in the different OS! workshops (e.g. professional groups, roles, management), and how did you experience the composition of participants?	**Reach**
What motivated your organisation to work with organisational health literacy and to participate in OS!?	**Adoption**
Which elements or characteristics of OS! made it attractive to your organisation?	**Adoption**
How did OS! unfold in your organisation, starting with the first workshop?	**Implementation**
What was your role in the OS! process?	**Implementation**
How did you experience participating in OS!? Please provide examples	**Implementation**
Was there anything that was easy to engage with during OS!?	**Implementation**
Was there anything that was more difficult or challenging?	**Implementation**
What was your experience with the reflection questions used in the first OS! workshop?<br>- How did these questions contribute to reflection and understanding of the concept of health literacy?	**Implementation**
OS! included several domains for assessing organisational health literacy. How did you experience working with these domains in relation to evaluating and improving your organisation's health literacy?<br>- Were there any domains that worked particularly well or less well? Please explain why.<br>- How did you experience the scoring system used in OS!?<br>- How was the scoring system subsequently used in your organisation?	**Implementation**
Was anything done differently during OS! than described in the OS! guide in order to better fit your organisation? (This question was only for facilitators)	**Implementation**
Did your understanding of health literacy change after completing OS!? If so, how?	**Effectiveness**
Do you think the improvement ideas generated through OS! could have arisen in other ways without participating in OS!?	**Effectiveness**

What came out of the OS! process in your organisation? Please provide concrete examples (e.g. initiatives, working groups, or action plans).	**Effectiveness**
How has your organisation worked with these outcomes after completing OS!?	**Maintenance**
Has OS! led to any changes in your organisation? Please provide concrete examples.	**Maintenance**
How have you experienced these changes?	**Maintenance**
After completing OS!, what initiatives has your organisation taken to ensure that organisational health literacy remains relevant and up to date?	**Maintenance**
What challenges or barriers have you encountered in trying to maintain the changes initiated through OS!?	**Maintenance**
How does your organisation measure or evaluate the effects of the organisational changes resulting from OS!?	**Maintenance**
What advice would you give to other organisations who are considering implementing OS!?	**Cross RE-AIM dimension**
What do you think worked well in OS!?	**Cross RE-AIM dimension**
Which aspects of OS! do you think could be improved?	**Cross RE-AIM dimension**
Do you have any specific suggestions for how the OS! could be made more effective or relevant?	**Cross RE-AIM dimension**
Is there anything else you would like to share about your experience with OS! that could help improve it in the future?	**Cross RE-AIM dimension**

Table S2: Overview of questionnaire items from workshops 1 and 2 used in the feasibility study, mapped to RE-AIM dimensions

Item no.	Question (English translation)	Response format*	RE-AIM dimension
**Q1**	*I had knowledge of health literacy before today's workshop*	1–5 Likert	**Context**
**Q2**	*I have gained an increased understanding of individual health literacy*	1–5 Likert	**Effectiveness**
**Q3**	*I have gained an increased understanding of organisational health literacy*	1–5 Likert	**Effectiveness**
**Q4**	*The workshop prompted relevant reflections and professional discussions*	1–5 Likert	**Effectiveness**
**Q5**	*The workshop led to concrete suggestions for improvements in our practice*	1–5 Likert	**Effectiveness**
**Q6**	*It is clear to me that working with organisational health literacy is important in our organisation*	1–5 Likert	**Adoption**
**Q7**	*Working with organisational health literacy fits with other activities in our organisation.*	1–5 Likert	**Adoption**
**Q8**	*I was engaged in the group discussions during the workshop.*	1–5 Likert	**Implementation**
**Q9**	*The questions we worked with in the groups were clear and easy to discuss*	1–5 Likert	**Implementation**
**Q10**	*There was sufficient facilitation during the workshop*	1–5 Likert	**Implementation**
**Q11**	*The scoring system (red–yellow–green) helped promote discussions in our group.*	1–5 Likert	**Implementation**
**Q12**	*Do you have any other reflections or comments regarding the workshop*	Open-ended	**Implementation**
**Q13**	*What barriers do you see to continuing work with organisational health literacy*	Open-ended	**Maintenance**
**Q14**	*What is needed to overcome these barriers*	Open-ended	**Maintenance**

***Items 1–11 were rated on a five-point Likert scale: 1 = Not at all; 2 = To a limited extent; 3 = To some extent; 4 = To a high extent: 5 = To a very high extent

Table S3: Overview of questionnaire items from workshops 3 used in the feasibility study, mapped to RE-AIM dimensions

Item no.	Question (English translation)	Response format*	RE-AIM dimension
**Q1**	*The right people participated in today's workshop*	1–5 Likert	**Reach**
**Q2**	*Were there any people who were missing – if yes who?*	Yes/no Open-ended	**Reach**
**Q3**	*The workshop helped us reach agreement on concrete initiatives we will continue to work on*	1–5 Likert	**Effectiveness**
**Q4**	*The OHL-Act workshops have strengthened collaboration between management and practice regarding organisational health literacy*	1–5 Likert	**Effectiveness**
**Q5**	*It was important for me to participate in today's workshop*	1–5 Likert	**Adoption**
**Q6**	*I felt that my viewpoints were heard during the workshop*	1–5 Likert	**Implementation**
**Q7**	*The structure of the workshop supported relevant prioritisation of organisational health literacy initiatives*	1–5 Likert	**Implementation**
**Q8**	*Do you have any other reflections or comments regarding the workshop?*	Open-ended	**Implementation**
**Q9**	*Based on the workshops, I wish to participate in the ongoing work on organisational health literacy*	1–5 Likert	**Maintenance**
**Q10**	*It is clear to me how we can move forward with organisational health literacy in the short term*	1–5 Likert	**Maintenance**
**Q11**	*It is clear to me how we can move forward with organisational health literacy in the long term*	1–5 Likert	**Maintenance**

*****Items 1–7 and 9-11 were rated on a five-point Likert scale: 1 = Not at all; 2 = To a limited extent; 3 = To some extent; 4 = To a high extent: 5 = To a very high extent

Table S4: Overview of OHL-Act

Workshop	Objective	Matching tool	Duration
**Workshop 1 - Reflection**	Participants' reflections on the concept of health literacy and its application in their local context.	Reflection exercise consisting of five open-ended questions.	1–2 hours, depending on participants' familiarity with the concept of health literacy.
**Workshop 2 - Self-Assessment**	Self-assessment of local strengths and barriers in organisational health literacy, accompanied by the development of concrete ideas for improvement.	Self-assessment tool comprising 20 discussion points organised across eight key domains for organisational health literacy. Guided by concrete prompts and examples, participants engage in facilitated group reflection to identify strengths and areas for improvement in current practices and to generate locally relevant ideas to improve organisational health literacy.<br><br>The eight domains are (1) Leadership & Culture; (2) Competencies; (3) Process & Practice; (4) Involvement; (5) Access; (6); Communication; (7) Vulnerability & High Risk; (8) Monitoring & Evaluation	Minimum 4 hours, depending on the number of participants and depth of discussion.
**Workshop 3 - Prioritisation**	Prioritisation of improvement ideas to inform and guide improvement planning.	Prioritisation tool for evaluating improvement ideas within each of the eight domains, based on importance, urgency, and required resources.	3–4 hours, depending on the number of participants.
**Future planning**	To ensure that the process moves beyond workshops by supporting the development of a concrete implementation plan, translating improvement ideas into actions with organisational commitment.	A tool to support the organisation in developing an action or improvement plan for organisational health literacy responsiveness.	Should be conducted at the management level to ensure sufficient resources for implementation
**Support material**	**Description**		
Facilitation guide	Comprehensive support material created to help apply the OHL-Act approach in various organisations and by professionals without a scientific background.		

Table S5: Detailed description of OHL-Act

<table>
<tr><th colspan="2">OVERVIEW OF THE INTERVENTION (OHL-ACT)</th></tr>
<tr><td colspan="2">The OHL-Act intervention is a structured organisational reflection and action-planning process designed to support health care organisations in identifying and prioritising improvements related to organisational health literacy (OHL). The intervention does not introduce predefined practice changes or prescribe specific solutions. Instead, it provides a systematic framework through which organisations examine their existing practices and generate locally tailored improvement initiatives. The full OHL-Act implementation guide is available in Danish [1] and an English translation is under preparation.

OHL-Act consists of three sequential workshops involving multidisciplinary staff and leadership representatives. The workshops are typically delivered within a period of 4-12 weeks.

Workshop 1 introduces the concept of health literacy and OHL and facilitates structured reflection on how current organisational practices in relation to these concepts may create barriers or demands for service users.
Workshop 2 engages participants in a systematic self-assessment of organisational practices across eight predefined OHL domains. Working in groups, participants identify existing strengths and areas for improvement in the organisation practices and then identify concrete proposals for organisational change.
Workshop 3 focuses on prioritising the proposed initiatives using explicit criteria related to perceived importance, implementation timeframe, and resource requirements.

The process concludes with the development of an action plan specifying selected initiatives, responsible actors, and proposed timelines. The content of the initiatives is determined locally by the participating organisation. OHL-Act therefore functions as a structured organisational learning and decision-support process rather than a direct behavioural or clinical intervention.

To allow smooth local implementation, many parts of the OHL-Act is designed to be locally adaptable. This adaption may affect the number and composition of participants, the facilitation and design of workshop elements and the condensation of output. However, mandatory criteria for OHL-Act implementation regarding workshop content, staff involvement, action plan development and leadership engagement is included in the implementation guide.</td></tr>
<tr><th colspan="2">PRE-IMPLEMENTATION PHASE</th></tr>
<tr><td>Leadership Engagement</td><td>Prior to initiation, formal endorsement from organisational leadership is required. Leadership commitment includes allocation of protected time for staff participation and agreement to consider the resulting action plan.</td></tr>
<tr><td>Facilitator Appointment</td><td>OHL-Act is designed to be delivered by a process facilitator with some knowledge of health literacy and experience in process facilitation. The facilitator is responsible for guiding workshop sessions and discussions, maintaining the structured progression of the process, and</td></tr>
</table>

	analysing and condensing outputs between workshops. The facilitator may be internal or external to the organisation, but some level of local representation is needed in the planning and implementation of the OHL-Act.
Participant Composition	OHL-Act is intended to involve multidisciplinary staff representing relevant professional roles within the organisation, alongside leadership representatives where appropriate. Broad representation is expected to support diverse perspectives and promote shared organisational ownership of identified initiatives.
**IMPLEMENTATION PHASE**	
**Workshop 1 - Reflection**	The purpose of workshop 1 is to create a shared understanding of the concepts of health literacy and OHL and generate reflection about how the organisation meets citizens with varying levels of health literacy.<br><br>The workshop includes a learning session based on a presentation by the facilitator (template available in Danish) and a reflection session based on group discussions of five open-ended questions (available in the facilitators guide).<br><br>The workshop does not necessarily require documentation. However, if workshops 1 and 2 are not carried out in continuation of each other, it is recommended to use a condensation of key themes from group discussions as part of the introduction to workshop 2.
**Workshop 2 –**<br><br>**Self-assessment**	The purpose of workshop 2 is to evaluate the organisation across eight core areas of OHL, using OHL-Act's systematic self-assessment tool (available in the facilitators guide).<br><br>The tool consists of eight sections corresponding to the eight core areas. The eight core areas are clarified using short descriptions at the top of each section. Across the eight core areas, the tool consists of 20 discussion prompts (2-3 per core area), each of which includes a headline, a question and a range of examples of relevant practices. The tool guides participants to assess organisational strengths ("what do we do well?") and weaknesses ("what could we improve?") relating to the specific question and then bring about discussions on specific improvement ideas ("what (specifically) can we do?").<br><br>The tool is applied in group sessions with 5-8 participants per group, and the facilitator is guided in the process of forming the groups based on participant's functions and relations. It is recommended. that leaders are placed in their own group to ensure honest and open discussions unaffected by obvious power relations. It is further recommended that when practically feasible each group is assigned a maximum of four out of the eight core areas to assess in order to ensure time and engagement throughout the exercise, but also that at least two groups assess each of the eight core areas to ensure several perspectives on each.<br><br>It is recommended that a time-holder and recording secretary is assigned to each group to support the process and documentation. This person should not be part of the discussions. Documentation includes notes on the group reflections and meticulous recording of the improvement ideas in as much detail as possible.

**Inter-Workshop Consolidation Phase**	After Workshop 2, the facilitator (sometimes with the help of a larger team) is responsible for the initial sorting of improvement ideas. This step is intended to prepare the material for structured prioritisation rather than to influence content Consolidation generally includes merging ideas that are similar and discarding ideas that are either unrelated to OHL or too vague to be included in the prioritisation workshop. Such imprecise ideas often take the form of dream scenarios or ideal end-goals. These can be used in Workshop 3 as part of the general scene-setting, but they should not be treated as concrete suggestions for prioritisation.
**Workshop 3 - Prioritisation**	The purpose of workshop 3 is to select the most important ideas for improvement and convert them into elements in an action plan. The workshop can be carried out in several ways, and the facilitator guide includes three formats to be inspired by.<br><br>In any format, the workshop guides participants through systematic assessment and decision-making choosing among the many improvement ideas. The prioritisation is based on three criteria: The importance of the idea (to invoke relevant organisational changes), the resource demand of the idea (if implemented) and the timeline of a potential implementation (short-term or long-term). It is recommended that both short-term and easily achievable ideas and more long-term comprehensive ideas are prioritised in order to support substantial change while also allowing for “quick wins” that may support a positive attitude towards the OHL work in general.<br><br>Participants in workshop 3 ideally include representatives from all groups in workshop 2 and the relevant leaders and decision-makers, that are needed to ensure that prioritised ideas are feasible and supported by management. It is recommended that staff representatives outnumber the leaders and decision-makers.<br><br>Workshop 3 results in a list of prioritised ideas readily inputted into an action plan. It is recommended to include a session in the workshop for consolidation of the endorsement of the prioritised ideas and general agreement on the way forward including who is responsible for the implementation of each part of the action plan.
**POST-IMPLEMENTATION PHASE**	
As the OHL-Act does not define what initiatives to develop and prioritise, the approach cannot give directions regarding specific implementation of improvement ideas. However, the facilitator guide does give general recommendations regarding the phase following the workshops which are summed up below.	
**Sharing of results**	Any staff or leaders not participating in the OHL-Act should be informed about the process and results to ensure transparency, create shared understanding, prepare for upcoming changes, and strengthen engagement.<br><br>There may also be more overall managing bodies or external partners, that can benefit from information on the process and a change to contribute to the action plan.

**Action plan**	If it has not been done at workshop 3, the facilitator or any other person appointed to the task, should draft an initial action plan based on the prioritised improvement ideas. The plan must be finalised together with the relevant management to ensure leadership support and sufficient resources for implementation. In many cases, it is beneficial to establish working groups, change agents or other work forums based on employees' engagement and competencies to drive specific improvement initiatives.
**Implementation**	As evident from the above, the organisation's expectations should be realistic and aligned with its existing capacity and workflows.<br><br>It is recommended that employees are actively involved in the implementation to maintain ownership and support for the change process. Implementation can be further strengthened by choosing quick wins, making early successes visible, setting a long-term goal with a timeline, ensuring regular follow-up at management and staff meetings, and providing the necessary resources and support.<br><br>Engaged and involved leadership is essential to ensure that improving OHL becomes part of ongoing development rather than a temporary effort.
**Monitoring**	Regular follow-ups - such as repeating OHL-Act processes or conducting status reviews - help evaluate and strengthen the efforts over time. The frequency depends on the goals and the initiatives implemented. It is recommended that concrete goals are set for each initiative and that it is regularly assessed whether planned activities have actually been completed as intended.

Figure S1: Response frequencies (%) for feasibility-related items from workshops 1 and 2 (N = 21)

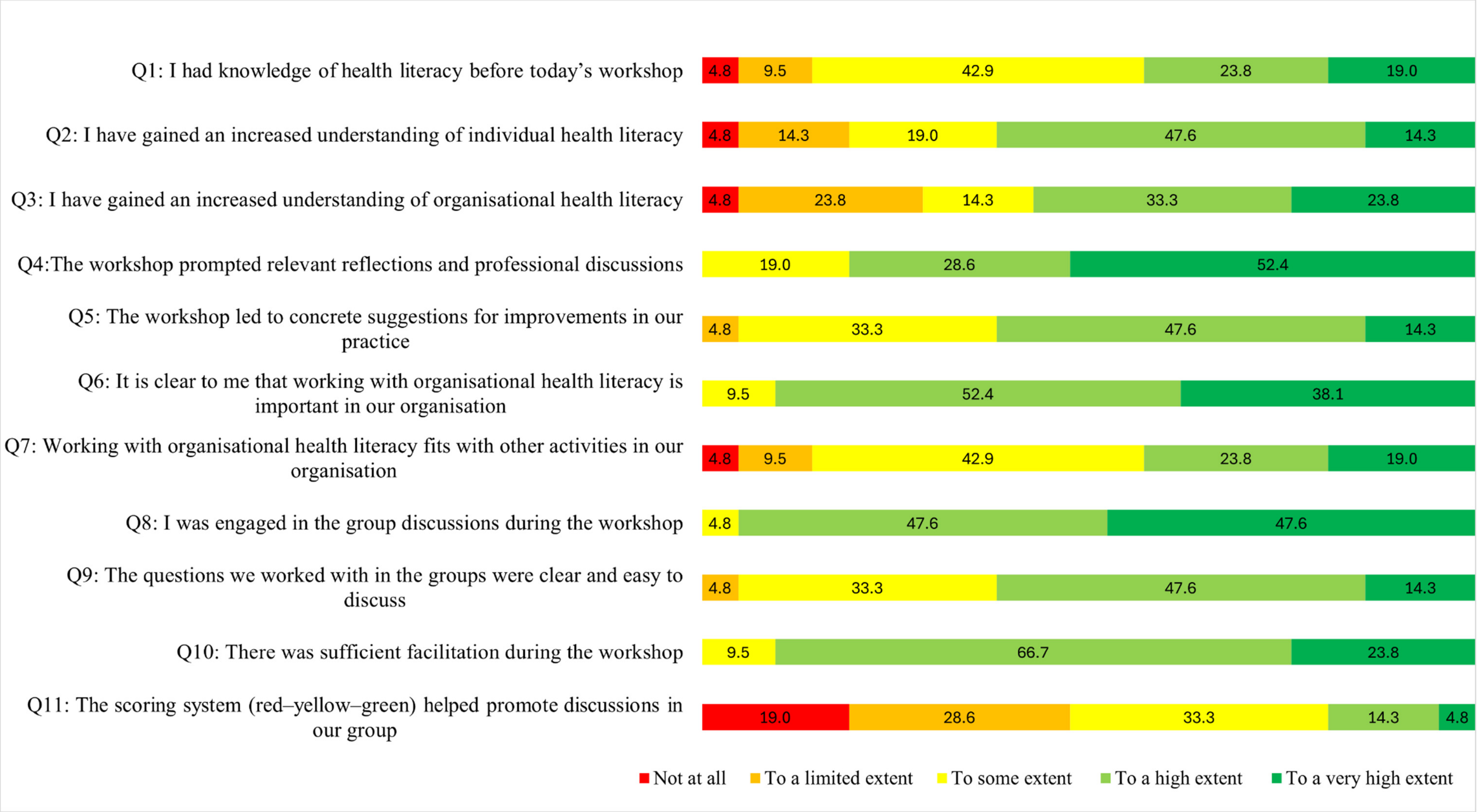

Three open-ended questions were also included: (Q12) "Do you have any other reflections or comments regarding the workshop?" (Q13) "What barriers do you see in relation to continuing work on organisational health literacy?", and (Q14) "What is needed to overcome these barriers?". Response rates were 52.4%, 47.6%, and 28.6%, respectively.

For organisational and practical reasons, workshops 1 and 2 were combined and conducted on the same day.

Figure S2: Response frequencies (%) for feasibility-related items from workshop 3 (N = 10)

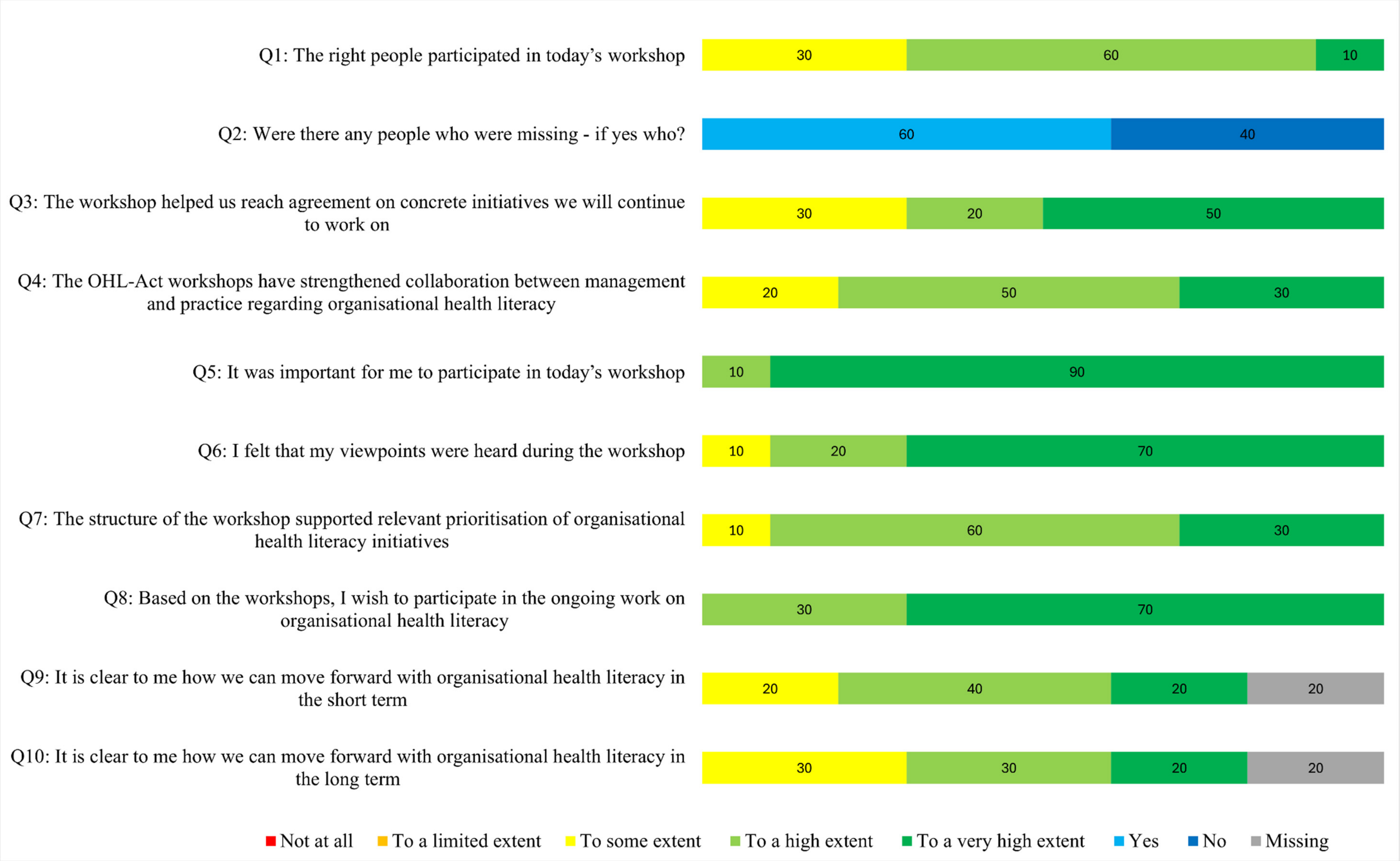

One open-ended questions were included: (Q11) "Do you have any other reflections or comments regarding the workshop?", which was answered by 30% of participants

References

1. Renneberg CK, Rasmussen ASD, Maindal HT, Aaby A. OS - Et redskab til udvikling af organisatorisk sundhedskompetence [OHL-Act - a tool for organisational health literacy development]. København S; 2025 Sep. Report. Available from: www.sst.dk
